# Histopathological changes in the liver of tree shrew (Tupaia belangeri chinensis) persistently infected with hepatitis B virus

**DOI:** 10.1186/1743-422X-10-333

**Published:** 2013-11-12

**Authors:** Ping Ruan, Chun Yang, Jianjia Su, Ji Cao, Chao Ou, Chengpiao Luo, Yanping Tang, Qi Wang, Fang Yang, Junlin Shi, Xiaoxu Lu, Linqun Zhu, Hong Qin, Wen Sun, Yuanzhi Lao, Yuan Li

**Affiliations:** 1 Department of Experimental Pathology, Guangxi Cancer Institute (Guangxi Tumor Hospital), Nanning 530021, China; 2 Graduate School of Guangxi Medical University, Nanning 530021, China; 3 School of Pharmacy, Shanghai University of Traditional Chinese Medicine, Shanghai 201203, China

**Keywords:** Tree shrew (*Tupaia*), Hepatitis B virus, Histopathological change

## Abstract

**Background:**

An animal model for HBV that more closely approximates the disease in humans is needed. The tree shrew (*Tupaia belangeri*) is closely related to primates and susceptible to HBV. We previously established that neonatal tree shrews can be persistently infected with HBV *in vivo*, and here present a six year follow-up histopathological study of these animals.

**Methods:**

Group A consists of six tree shrews with persistent HBV infection, group B consists of three tree shrews with suspected persistent HBV infection, while group C consists of four tree shrews free of HBV infection. Serum and liver tissues samples were collected periodically from all animals. HBV antigen and HBV antibodies were detected by ELISA and/or TRFIA. HBV DNA in serum and in liver biopsies was measured by FQ-PCR. Liver biopsies were applied for general histopathologic observation and scoring, immunohistochemical detections of HBsAg and HBcAg, and ultrastructural observation with electron microscope technique.

**Results:**

Hydropic, fatty and eosinophilic degeneration of hepatocytes, lymphocytic infiltration and hyperplasia of small bile ducts in the portal area were observed in group A. One animal infected with HBV for over six years showed multiple necrotic areas which had fused to form bridging necrosis and fibrosis, and megalocytosis. The hepatic histopathological scores of group A were higher than those of group B and C. The histopathological score correlated positively with the duration of infection.

**Conclusions:**

Hepatic histopathological changes observed in chronically HBV-infected tree shrews are similar to those observed in HBV-infected humans. The tree shrew may represent a novel animal model for HBV infection.

## Background

Hepatitis B virus (HBV) infection is a global health problem. At least 2 billion people, one third of the world’s population, are currently infected with HBV worldwide, including 30 million people in China [1,2]. About 6% of the world’s population (378 million) are chronic carriers of HBV, and approximately 1 million people die each year from complications such as cirrhosis and hepatocellular carcinoma (HCC) arising from HBV infection [1]. Although a prophylactic vaccine against HBV is currently available [3], the efficacy is limited within individuals previously infected with HBV. This immunization regimen does not prevent HBV carriers from developing HCC and other end-stage liver diseases. Improvements in our understanding of the mechanisms underlying HBV persistence *in vivo* may lead to new approaches for treating chronic hepatitis B or preventing infection from progressing to life-threatening lesions. A suitable animal model of persistent HBV-infection, is thus in urgent demand.

HBV infection is almost entirely limited to human and chimpanzee providing an obstacle to the establishment of an animal model of HBV-infection. Alternative models have been pursued, including woodchuck, ground squirrel or Peking duck hepadnavirus infections; however the hepatic pathogenesis mediated by these viruses is not equivalent to human infection with HBV [4,5]. The chimpanzee model of HBV infection is useful, but as an experimental subject, it is relatively rare, expensive and inconvenient to manage.

The Tree shrew (*Tupaia*) is phylogenetically much more closely related to humans [6,7], and is susceptible to a variety of human viruses [8,9]. Walter et al. have successfully infected tree shrew extracorporeal hepatocytes with HBV, and established replication of HBV in these hepatocytes [10-12]. Our previous studies suggested that persistent HBV infection can be established in neonatal tree shrews [13,14]. Continuing observation of those HBV-infected tree shrews for several years may illuminate the nature of the infection. In this study, we focused on hepatic histopathological changes in these experimentally HBV-infected animals.

## Results

### General information

The criteria based on which “confirmed chronic-infection” and “suspected chronic-infection” were defined have been previously described [14]. “Confirmed chronic infection” indicates infection of animals in which serum HBsAg is detectable throughout the 48 weeks after inoculation, in addition to that HBV DNA tests positive in either serum or liver biopsies. “Suspected chronic infection” indicates infection of animals in which two of the previously described markers are detectable on at least two occasions within the 48 weeks after inoculation.

According to the HBV infection status, the tree shrews were divided into three groups: group A consists of six animals that were confirmed as chronically infected; group B consists of three animals that were suspected to be chronically infected, while group C consists of four normal animals that were not inoculated with HBV. Table 1 describes the general information of the tree shrews used in this study.

**Table 1:** Tree shrews assigned to groups based on HBV infection status

**Group**	**Experimental no.**	**Gender**	**Age (weeks)**	**Weeks after inoculation**
A (n = 6)	A1	♀	324	324
	A2	♂	89	89
	A3	♀	275	275
	A4	♂	221	221
	A5	♂	149	149
	A6	♂	218	218
B (n = 3)	B1	♀	229	229
	B2	♀	221	221
	B3	♀	171	171
C (n = 4)	C1	♂	79	-
	C2	♂	79	-
	C3	♂	64	-
	C4	♂	64	-

### Markers of HBV infection in inoculated tree shrews

As described in Table 2, the tree shrews assigned to group A were serum HBsAg-positive until the last inspection (1.7-6.2 years after inoculation). The sera obtained from four of these animals were simultaneously HBeAg and HBcAb positive, and two animals tested HBeAb and HBcAb positive simultaneously. Within group B, the serum of one animal tested weakly positive for HBsAg and two tested positive for HBeAb, while all the three animals in this group were serum HBcAb- and HBsAb-positive simultaneously. All samples from the four animals within group C tested negative for all the HBV-markers mentioned above.

**Table 2:** **HBV immunological markers in tree shrew serum samples**^**a**^

**Group**	**Experimental no.**	**HBsAg**^**a**^	**HBsAb**	**HBeAg**	**HBeAb**	**HBcAb**	**HBsAg**^**a **^**(ng/ml)**
A (n = 6)	A1	+	-	+	-	+	450.6
	A2	+	-	-	+	+	797.85
	A3	+	-	+	-	+	704.57
	A4	+	-	+	-	+	615.88
	A5	±	-	-	±	+	0.95
	A6	+	-	+	-	+	711.14
B (n = 3)	B1	±	+	-	-	+	0.03
	B2	-	+	-	+	±	0
	B3	-	+	-	+	+	0
C (n = 4)	C1	-	-	-	-	-	0
	C2	-	-	-	-	-	0
	C3	-	-	-	-	-	0
	C4	-	-	-	-	-	0

^a^The results detected by ELISA were expressed as “+” (positive), “-” (negative) or “±” (between positive and negative), while by TRFIA were expressed as ng/ml.

The results of HBV DNA determination in serum and in liver biopsies are shown in Table 3. Among the six animals in group A, the copy number of HBV DNA in serum of five animals was above 10^3^. The copy number of HBV DNA in the liver tissues of group A animals were significantly higher than that of group B (*P* = 0.039) and group C animals (*P* = 0.011), while the difference in copy number between group B and group C is not statistically significant (*P* = 0.400).

**Table 3:** Results of determination on HBV DNA in serum and tissue samples

**Group**	**Experimental no.**	**HBV DNA in serum (Copies/ml)**^**a**^	**HBV DNA in liver**		
			**Copies/μg liver DNA**^**b**^	**Copies**^**c**^	***P-value***
A (n = 6)	A1	2.63 × 10^6^	1.83 × 10^8^	1.12 ± 0.99 × 10^8^	
	A2	5.22 × 10^3^	1.27 × 10^8^		
	A3	8.74 × 10^6^	5.17 × 10^8^		
	A4	1.09 × 10^6^	2.64 × 10^8^		
	A5*	-	7.61 × 10^4^		
	A6	2.42 × 10^5^	4.36 × 10^7^		
B (n = 3)	B1	-	9.58 × 10^5^	3.56 ± 5.22 × 10^5^	0.039^d^
					0.400^b^
	B2	-	3.91 × 10^4^		0.400^e^
	B3	-	7.04 × 10^4^		
C (n = 4)	C1	-	6.35 × 10^4^	5.06 ± 2.18 × 10^4^	0.011^d^
	C2	-	1.90 × 10^4^		
	C3	-	6.63 × 10^4^		
	C4	-	5.34 × 10^4^		

^a^HBV DNA copy number ≥10^3^ in serum is defined as a positive; ^b^HBV DNA copy number ≥10^5^ in liver tissue is defined as a positive; ^c^Results are shown as mean ± SD; ^d^Compared with group A; ^e^Compared with group C.

*: This animal had showed 1.1 × 10^7^ copies/ml in serum, 3.73 × 10^5^ and 1.82 × 10^8^ copies/μg in liver at the time points of 60- and 72-Weeks after inoculation.

**Figure 1:**
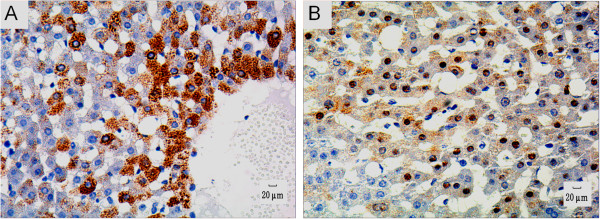
**Immunohistochemical detection of HBV markers in liver. A**. Immunohistochemical staining highlighting the presence of HBs antigen within the cytoplasm of some hepatocytes, 200×. **B**. Immunohistochemical staining highlighting the presence of HBc antigen within the cytoplasm and/or nucleus of some hepatocytes of some hepatocytes, 200 ×.

Immunohistochemical staining of liver biopsies revealed a pattern of sporadically or focally distributed HBsAg-positive hepatocytes within all six animals in group A. HBsAg-positive cells were distributed predominantly around central veins, manifesting as yellowish-brown granules located mainly in the cytoplasm (Figure 1A). In all animals except one (83.3%) we detected HBcAg-positive hepatocytes. The HBcAg-positive hepatocytes contained yellowish-brown granules, mainly located in the nuclei but also observed in the cytoplasm (Figure 1B). All the liver biopsies from animals in group B and group C tested negative for HBsAg and HBcAg staining.

### Histopathological examination of the liver

**Figure 2:**
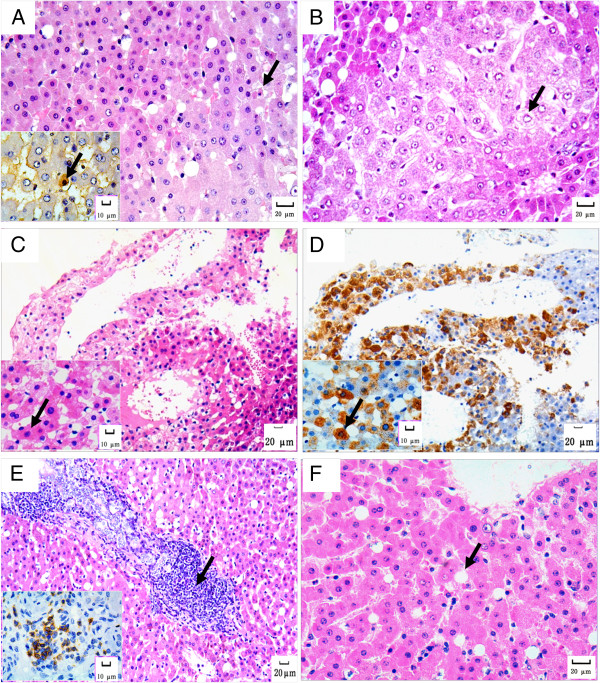
**Histopathological changes in the liver of tree shrew with persistent HBV-infection. A**. Hydropic degeneration, fatty degeneration and acidophilic degeneration (arrow) in some hepatocytes. HE staining, 200×. The insert image visualizes the apoptotic cells (arrow) confirmed by TUNEL assay. TUNEL staining, 400×. **B**. Ballooning degeneration in hepatocytes (arrow). HE staining, 200×. **C**. Ground-glass-like hepatocytes in animal A1 who was HBV-infected for more than 6 years (arrow). HE staining, 100× and 400× respectively. **D**. The ground-glass-like hepatocytes were proved to be HBsAg-positive (arrow). Immunohistochemical staining for HBsAg, 100× and 400× respectively. **E**. Portal tracts were infiltrated with densely packed lymphocytes (arrow), accompanied with bile duct proliferation. HE staining, 100×. The insert image visualizes the CD3-positive lymphocytes. Immunohistochemical staining for CD3, 400×. **F**. Minor histopathological changes in one animal of group B. HE staining, 200 ×.

All animals in group A showed liver tissue injury at different degrees, characterized by sporadically or diffusely distributed hepatocellular hydropic degeneration, fatty degeneration and acidophilic degeneration. Hepatocytes undergoing acidophilic degeneration were sporadically distributed, characterized by reduced cell size and concentrated appearance, with strengthened cytoplasmic acidophilia and small and dark nuclei. TUNEL staining showed that these hepatocytes were apoptotic cells (Figure 2A). Round and red-stained small bodies without nuclei, so called “acidophilic bodies”, were sometimes observed among these cells. The hepatocytes undergoing hydropic degeneration were pale, with enlarged size and sparse cytoplasm containing fine granules. The cytoplasm of the hepatocytes with severe swelling was lighter in color and even almost transparent, termed “ballooning degeneration” (Figure 2B). In addition we observed scattered or focally distributed ground-glass hepatocytes, the cytoplasm of which contained obviously acidophilic granules, appearing as non-transparent and ground-glass-like (Figure 2C). Serial sections of these samples were stained immunohistochemically, demonstrating that ground-glass hepatocytes were HBsAg-positive (Figure 2D). Additional histopathologic changes were also observed, including focal hepatocellular necrosis, lymphocytic infiltration dominated by CD3-positive (rather than PAX5-positive) T-lymphocytes, with formation of lymphoid follicles and biliary proliferation in portal areas (Figure 2E). Aside from minor degeneration of a minority of hepatocytes, no obvious histopathological changes were observed in the liver biopsies from the animals in both groups B and C (Figure 2F).

**Figure 3:**
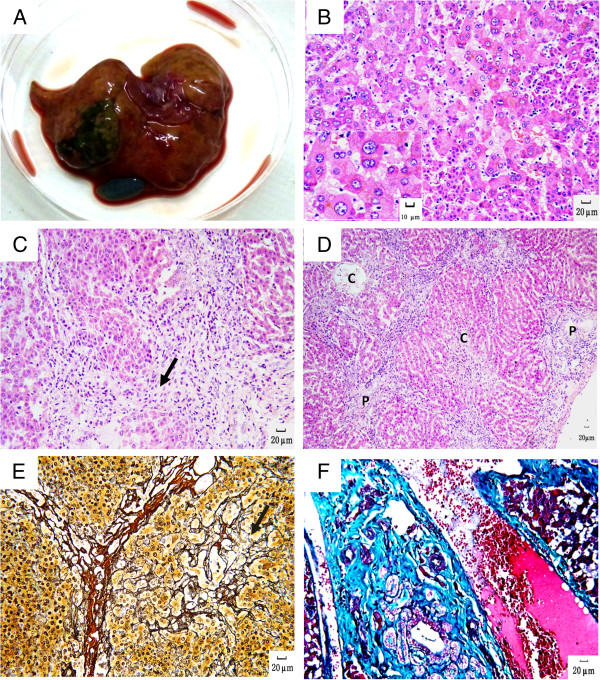
**Gross and histological images of tree shrew no. A1. A**. The surface of the liver is coarsely nodular with multifocal mottled tan and red areas. **B**. Megalocytosis. HE staining, 200× and 400× respectively. **C**. Piecemeal necrosis (arrow) characterized by lymphocytic infiltration extends from portal areas and disrupts the limiting plate of hepatocytes undergoing necrosis. HE staining, 100×. **D**. Bridging necrosis as a streak of hepatic necrosis extends from central vein (C) to portal tracts (P). HE staining, 40×. **E**. Reticular fiber collapses and intertwines to form a cluster (arrow) in limiting plate. Gomori’s silver staining, 200× and 400× respectively. **F**. There is increased collagen in portal areas. Masson staining, 200×.

The most severe histopathological changes in the liver were found in one animal (A1) in group A who survived the longest post-inoculation, for more than 6 years until the end of this study. Under gross examination, the liver of this animal was characterized by an unsmooth surface, granular protuberances, yellow and red patchy lesions on section (Figure 3A). Microscopically, large numbers of hepatocytes exhibited diffuse hydropic or fatty degeneration. Binucleate or even multinucleate hepatocytes were also observed frequently, these cells displayed enlarged size and a normal ratio of nucleus to cytoplasm (Figure 3B), i.e., megalocytosis, similar to “large cell dysplasia” observed in human patients with chronic hepatitis B. Meanwhile, further lessons were also observed such as confluent lytic necrosis inside lobules, piecemeal necrosis in limiting plate areas, and structural damage in limiting plates (Figure 3C). Confluent lytic necrosis of hepatocytes was observed between the portal tract and central vein and between neighboring portal tracts, which formed bridging necrosis and bridging fibrosis (Figure 3D). Reticular fiber staining revealed that the scaffolds of reticular fiber had collapsed, and that the fibers had intertwined and surrounded hepatocytes in the limiting plate area and formed irregular hepatocyte-clusters that were mostly composed of survived or regenerated hepatocytes, and were without normal lobular architecture (Figure 3E). Masson staining revealed notable proliferation of collagenous fiber bundles in portal tracts (Figure 3F).

The Liver histological status was evaluated semiquantitatively, according to the Knodell histology activity index scoring system which grades necroinflammation and fibrosis on a scale of 0 to 22, including 0 to10 for the periportal and/or bridging necrosis, 0 to 4 for intralobular degeneration and focal necrosis, 0 to 4 for portal inflammation, and 0 to 4 for fibrosis [15].

**Figure 4:**
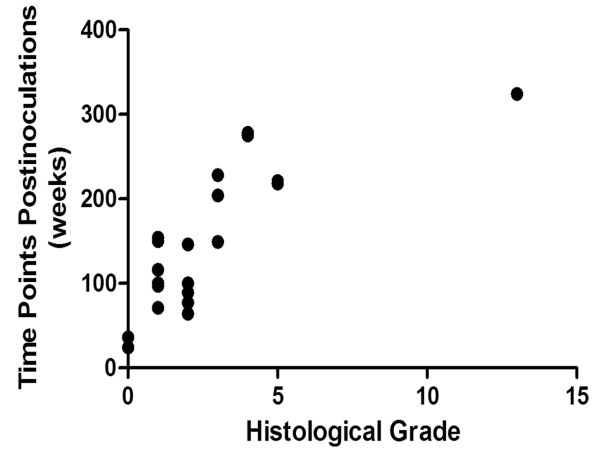
**Correlation between histopathological score and duration of HBV-infection (r = 0.808, *P*** **= 0.000).**

The histopathological scores of the last liver biopsies of the animals in each group are described in Table 4. The average score in group A, B and C was 5.33 ± 3.93, 1 and 0, respectively. The score of group A were significantly higher than those of group B (*P* = 0.018) and group C (*P* = 0.008), while the difference between the scores of group B and C was not statistically significant (*P* = 0.057). As shown in Figure 4, the histopathological score was correlated significantly with the duration of HBV infection (*P* = 0.000), but not with the titer of HBV DNA in liver tissue (*P* = 0.159).

**Table 4:** Histopathological score of the animals in each group and statistical analysis

**Group**	**Experimental no.**	**Periportal & bridging necrosis score**	**Intralobular degeneration & focal necrosis score**	**Portal inflammation score**	**Fibrosis score**	**Total score**	**Mean ± SD**	***P-value***
A (n = 6)	A1	3	4	3	3	13	5.33 ± 3.93	
	A2	0	1	1	0	2		
	A3	1	2	1	0	4		
	A4	1	2	1	1	5		
	A5	1	1	1	0	3		
	A6	1	2	1	1	5		
B (n = 3)	B1	0	1	0	0	1	1.00 ± 0.0	0.018^a^
	B2	0	1	0	0	1	0	0.057^b^
	B3	0	1	0	0	1		
C (n = 4)	C1	0	0	0	0	0	0.00 ± 0.00	0.008^a^
	C2	0	0	0	0	0		
	C3	0	0	0	0	0		
	C4	0	0	0	0	0		

^a^Compared with group A. ^b^Compared with group C.

### Hepatic ultrastructure

**Figure 5:**
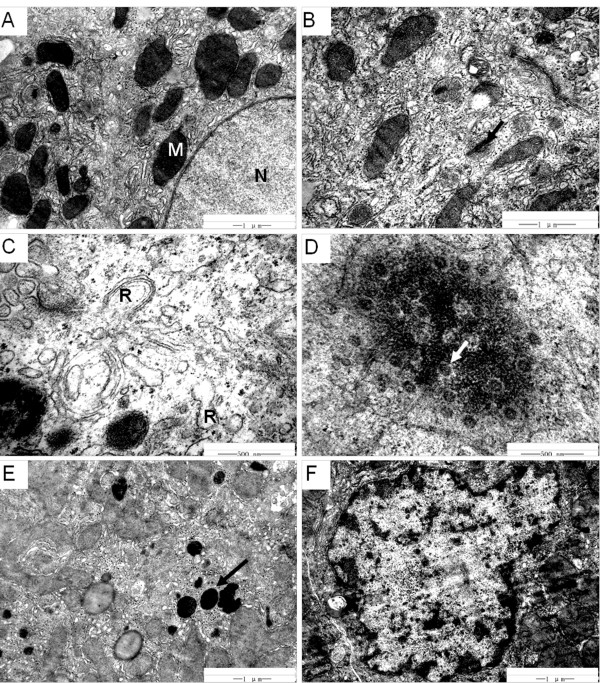
**Hepatocyte ultrastructure in persistent HBV infection of tree shrews under TEM. A**. Mitochondria were commonly swollen and enlarged, matrix electron density increased, and cristae were vague. M, mitochondria; N, nuclei. Osmic acid staining, 20000×. **B**. Electron dense rhabdoid crystalline inclusions (arrow) in mitochondria. Osmic acid staining, 30000×. **C**. Endoplasmic reticulum expanded or formed irregular vesicles with slight degranulation, and the expanded segments were surrounded by paired membranes, forming concentric lamellar bodies. R, reticulum. Osmic acid staining, 60000×. **D**. Suspected HBV granules in endoplasmic reticulum (arrow). Osmic acid staining, 60000×. **E**. The number of lysosomes (arrow) increased. Osmic acid staining, 20000×. **F**. Cell nuclei appeared irregular in shape and chromatin margination was observed. Osmic acid staining, 20000×.

Transmission electron microscopy was performed on sections of liver from animal A1 at the end of the study and demonstrated swollen hepatocellular surface microvilli, variable numbers and distribution of glycogen granules in the cytoplasm and variation in size of lipid droplets. Many mitochondria were swollen and enlarged with vague cristae and increased electron density in the matrix (Figure 5A). Highly electron dense rhabdoid crystalline inclusions were observed within the mitochondria (Figure 5B). The endoplasmic reticulum was expanded or formed irregular vesicles with slight degranulation, and part of the expanded endoplasmic reticulum were surrounded by paired membranes forming concentric lamellar bodies (Figure 5C). Suspected HBV granules were distributed as clusters and had a spherical appearance, and clear boundaries were observed in the cytoplasm and dilated endoplasmic reticulum (Figure 5D). The number of lysosomes increased, most of which were secondary lysosomes (Figure 5E), and some of which possessed lamellar residual bodies (myeloid bodies). Some cell nuclei were irregular in shape and chromatin margination was observed (Figure 5F).

## Discussion

Several animal models of HBV infection have been established, including the well-tested chimpanzee model and models utilizing alternative hepadnaviruses, but the hepatic lesions in these animal species are milder than those observed in chronic hepatitis B infection of humans [16-18]. Previously we reported that newborn tree shrews inoculated with HBV can develop chronic HBV-infection, and HBV can persist and replicate *in vivo* steadily for long time [14]. Subsequently, we have found that persistent HBV infection of tree shrews can induce histopathological changes in their liver that are quite similar to the changes in human cases.

Chronic HBV infection of humans is generally characterized as evolving in three major phases: immune toleration, immune activation and inactive carrier [19]. During the phase of immune tolerance, the immune system responses weakly against the HBV-infected hepatocytes, which allows HBV to survive and replicate. From this phase, most patients will progress to the phase of immune activation, with an elevated level of HBV DNA detected in the serum and active inflammation in liver tissue. After HBeAg is cleared from the body and anti-HBeAb is developed, most patients will progress to the phase of inactive carrier, characterized mainly by low or undetectable serum HBV DNA and reduced liver inflammation. The immune tolerance phase occurs more frequently in individuals who were infected with HBV perinatally. In this population this phase may last for decades, and the hepatic histopathological changes may become more evident as the host ages [20].

Previously we reported that only minimal histopathological lesions were found in the in the liver biopsies of these persistently HBV-infected tree shrews [14], but we have recently found that during prolonged infection this is not the case. This phenomenon can potentially be explained by the ability of neonatally HBV-infected tree shrews to remain in the immune tolerant phase for longer, without obvious changes in their liver tissues. With a longer period of observation we have found that at least some of these animals evolved into the immune-active phase, as the level of HBV DNA in liver tissue increased along with symptoms of injury in the liver tissues. The histopathological changes observed in the liver of animal A1 six years post-inoculation highlight the fact that conspicuous hepatic lessons can occur in persistently HBV-infected tree shrews.

Acute and chronic HBV-infection observed in the tree shrews in this study followed a similar course to human hepatitis B. Acute HBV infection in human adult is usually self-limiting. Serum HBsAg can be detected for only 4 ~ 10 weeks after infection, then HBsAb is generated and HBsAg disappears. This course of transient acute HBV-infection has also been reported in studies of adult tree shrews [10]. In contrast to adult tree shrews, six tree shrews that were neonatally inoculated with HBV in this study showed positive serum HBsAg over years. Considering the relatively short life span of the tree shrew (about 8 years), the duration of the HBV-infection markers in these animals is much longer than the established criteria for diagnosing chronic HBV-infection in human (6 months) [19]. Moreover, serum HBsAb was not detected in the six tree shrews who persistently showed HBsAg and HBV DNA in the serum and in liver, further supporting the diagnosis of chronic HBV infection.

A variety of pathologic changes were observed in each of the six chronically HBV-infected tree shrews, including hepatocellular degeneration and necrosis with mild or moderate periportal inflammation and fibrosis. The severity of these lesions often increased with the duration of infection. For example, significant piecemeal necrosis and bridging fibrosis were observed in animal A1 which was HBV-infected for longer than 6 years. It is generally recognized that piecemeal necrosis is a key milestone in progression of chronic hepatitis, and it can be used to distinguish chronic active hepatitis from chronic persistent hepatitis [21]. Whilst bridging fibrosis usually results from lobular necrosis which is then replaced by inflammatory fibrotic tissue, the latter appears as bridges linking the necrotic areas in portal spaces and/or in centrilobular areas.

Megalocytosis, characterized by large, atypical and multiple nuclei and abundant cytoplasm were also found in animal A1. The occurrence of such cells is believed to correlate with the risk of developing HCC [22,23]. In addition, the majority of chronic HBV-infected tree shrews in this study showed a relatively high level of cell proliferation markers such as Ki67, Cyclin D1 and P53, and HBx protein (a protein that has been associated with HCC in humans and the woodchuck) in liver tissues in comparison to the tree shrews without HBV infection (data not shown). It is reasonable to speculate, therefore, to further observation of these chronically HBV-infected tree shrews may demonstrate that HBV can act as an independent factor for hepatocarcinogenesis. However, compared to the carcinogenic effect of aflatoxin B1 (AFB1) alone or AFB1 plus HBV [24], the ability of HBV to induce HCC may be relatively weak and a longer period of infection may be required.

Additionally so-called “ground-glass” hepatocytes were found in half of the chronic HBV-infected tree shrews (3/6) in this study, and these cells were confirmed to be HBsAg-positive by immunohistochemical staining. The “ground-glass” hepatocytes are generally considered to be those cells containing a high concentration of HBsAg, and the number of such hepatocytes was reported to be inversely related to the activity of hepatitis [21]. With immunohistochemical staining, both HBsAg- and HBcAg-positive hepatocytes were detected in all chronically HBV-infected tree shrews (6/6) in this study. It is believed that the host’s immune response can eliminate antigen-containing cells, for example HBsAg- and HBcAg-positive hepatocytes should not be found in acute hepatitis B [21]. The presence of HBsAg- and HBcAg-positive hepatocytes, therefore, indicates that these tree shrews were in the chronic rather than the acute phase of HBV-infection. Concerning HBcAg, it is believed that its expression in the nucleus of hepatocytes indicates HBV replication [25], while its expression in the cytoplasm of hepatocytes indicated increased disease severity. Chu et al. [26] reported that the patients in whom HBcAg could be detected in both the nucleus and cytoplasm usually experienced more severe hepatocyte damage and higher titers of serum HBV DNA and HBeAg. Interestingly, a similar site of HBcAg expression in hepatocytes was observed in the tree shrews we studied. For example, there was no hepatic HBcAg expression in animal A5, who possessed low levels of HBV DNA in the serum and in liver simultaneously; While HBcAg expression in animal A2 was purely within the nucleus of hepatocytes, and hepatic histopathological changes were slight (scored 2); But the other four animals in group A (A1, A3, A4 and A6) expressing HBcAg in the nucleus and cytoplasm (as showing in Figure 1B), suffered much more obvious hepatic histopathological changes (scored 4 or more). These findings support the suggestion that the sites of HBcAg expression in hepatocytes relate to the titer of HBV DNA and the severity of hepatic pathological changes. A potential explanation for this observation is that the movement of HBcAg from the nucleus to the cytoplasm triggers cellular immunity, during which the hepatocytes with HBcAg expression are the targets of virus-specific T cells, activating inflammation in liver tissue [26].

There were significant ultrastructural findings within the liver. For example, the changes in the mitochondria might explain the hydropic and fatty degeneration of hepatocytes observed under light microscopy. The increased numbers of lysosomes and myeloid bodies possibly indicated hepatocellular injury, enhancement of autophagic activity and an increase of organelle residual bodies. The swelling of endoplasmic reticulum was characteristic of ultrastructural changes in hepatocytes induced by virus infection. In addition, suspected HBV granules with round appearance and cluster distribution were observed in the cytoplasm and expanded endoplasmic reticulum in some chronic HBV-infected animals such as A1.

## Conclusions

In summary, the histopathologic changes in the livers of tree shrews with chronic HBV infection provide further evidence that neonatally HBV-infected tree shrews can progress into chronic hepatitis, and both the clinical course and the hepatic histopathological changes that occur in these animals are fairly similar to those in human patients. The tree shrew model may provide an important tool for studying HBV-related diseases.

## Materials and methods

The study protocol was approved by the Ethical Committee of Guangxi Tumor Hospital in accordance with the guidelines issued by Chinese government, which conforms to the ethical guidelines of the 1975 Declaration of Helsinki.

### Animals

Animal experiments were carried out in accordance with the guidelines for care and use of laboratory animals issued by Chinese government. The tree shrews used in this study were descended from a population of wild tree shrews (Tupaia belangeri chinensis) originating from the Kunming Institute of Zoology, Chinese Academy of Science (Yunnan, China). Offspring resulting from artificial breeding were used in this study. The treatments of these animals, including HBV-inoculation and other experimental procedures, were previously described [14]. Animals were housed at the Laboratory Animal Center of Guangxi Medical University under monitoring of veterinarians.

### Blood and tissue sample collection

Blood samples were collected once every 4~12 weeks. The serum was tested immediately for HBV-infection markers: HBV surface antigen (HBsAg), HBV surface antibody (HBsAb), and HBV e antigen (HBeAg), HBV e antibody (HBeAb) and HBV core antibody (HBcAb), as well as HBV DNA. The remainder was stored at -80°C before use.

Liver biopsies were performed once every 6~12 months, under anesthesia with 1% pentobarbital and ketamine hydrochloride. A portion of each liver biopsy was fixed within 10% (v/v) neutral formalin and then paraffin embedded for routine histopathology observation and immunohistochemistry. When necessary, a portion of the biopsy was also fixed within 2.5% glutaraldehyde at 4°C for observation under transmission electron microscopy (TEM). The remainder of each biopsy sample was frozen immediately by immersion in liquid nitrogen then stored at -80°C before use.

### Detection of HBV directed antibodies in serum

The levels of HBV-directed antibodies present in animal serum samples was established by ELISA (Kehua Bio-engineering, Shanghai, China). HBsAg-positive specimens were then further analyzed quantitatively by time-resolved immunofluorescence analysis (TRFIA) (Xinbo Biotechnology, Suzhou, China). Kits were used according to the manufacturer’s instructions.

### Detection of HBV DNA in serum and in liver

The quantity of HBV DNA in serum and in liver samples was assessed by fluorescence quantitative polymerase chain reaction (FQ-PCR) (Kehua Bio-engineering Co. Shanghai, China) according to the manufacturer’s instructions as previously described [14], based on the procedures described by Lu et al. [27], or Cacciola et al. [28] respectively. The number of copies of serum-derived HBV DNA was calculated per ml while the liver-derived HBV DNA was calculated per μg liver DNA, based on the methods described by Cacciola et al. [28]. The threshold applied to classifying a tree shrew serum sample as positive for HBV DNA was identical to that for human samples, i.e. ≥ 10^3^ copies/ml is defined as a positive result by the kit’s manufacturer. While since no critical threshold for FQ-PCR measurement of hepatic HBV DNA has been established so far, we employed a cut-off value of ≥10^5^ copies/μg total liver DNA, referring to the results from detecting HBV-infected human (data not shown) and normal tree shrews.

### Histopathological and immunohistochemical analysis

Paraffin-embedded liver biopsies were stained with hematoxylin and eosin (H&E), and Gomori’s silver and Masson’s trichrome stains (Sinopharm Chemical Reagent Co., Ltd., Shanghai, China) were employed to visualize reticular and collagen fibers when necessary. All histological staining was performed in accordance with conventional procedures [29]. Intrahepatic HBsAg, HBcAg, CD3 and PAX5 were examined by Immunohistochemical assays, with the monoclonal antibodies of mouse anti-human HBsAg, HBcAg, CD3 or PAX5 respectively (Zhongshan Goldenbridge Biotech, Beijing, China) [13]. Apoptosis in liver tissues was detected by a terminal deoxynucleotidyl transferase dUTP nick-end labeling (TUNEL) assay using an In Situ Cell Death Detection Kit (Roche, Mannheim, Germany) according to the manufacturer’s instruction.

### Ultrastructure observation with transmission electron microscopy (TEM)

Transmission electron microscopy (TEM) was applied to observe the hepatic ultrastructure of animal A1 which was persistently infected with HBV for more than 6 years. A sample of its liver tissues obtained at the end of this study was fixed. The tissue was dehydrated with graded ethanol and embedded within epoxy resin (Sinopharm Chemical Reagent Co. Ltd, Shanghai, China). The 75 nm thick ultrathin sections were stained with methanolic uranyl acetate and lead citrate, then visualized by TEM (TECNAIG2, FEI Company, Hillsboro, Oregon, USA).

### Statistical analysis

Statistical analysis was conducted with SPSS 17.0 software (IBM, Chicago, USA). Animal groups were compared by non-parametric Mann–Whitney Test. The relationship between histological scores of the animals in group A with the duration of HBV infection, titer of HBV DNA in liver tissue, was analyzed by Spearman’s rank. The bilateral significance level was α = 0.05.

## Abbreviations

HBV: Hepatitis B virus; HBsAg: HBV surface antigen; HBsAb: HBV surface antibody; HBeAg: HBV e antigen; HBeAb: HBV e antibody; HBcAb: HBV core antibody; ELISA: Enzyme-linked immunosorbent assay; TRFIA: Time-resolved immunofluorescence analysis; FQ-PCR: Fluorescence quantitative polymerase chain reaction; TEM: Transmission electron microscope; HCC: Hepatocellular carcinoma; TUNEL: Terminal deoxynucleotidyl transferase dUTP nick-end labeling.

## Competing interests

Authors declare no financial or non-financial competing interests in relation to this manuscript.

## Authors’ contributions

PR carried out the pathology and molecular studies, performed the statistical analysis and participated in drafting the manuscript. CY carried out the animal experiments, participated in study design, pathology studies and drafting the manuscript. JJS, JC and CO participated in study design, animal experiments and pathology studies. CPL, YPT, QW, FY, JLS, XXL, LQZ, HQ and WS participated in the animal experiments and molecular studies. YZL made a substantial contribution to interpretation of data and was involved in manuscript revision and gave final approval of the version to be published. YL conceived of the study, designed and carried out animal experiments, and helped to draft the manuscript. All authors read and approved the final manuscript.

## Authors’ information

Ping Ruan and Chun Yang: co-first author.
